# The estrobolome: Estrogen‐metabolizing pathways of the gut microbiome and their relation to breast cancer

**DOI:** 10.1002/ijc.35427

**Published:** 2025-04-03

**Authors:** Ashley H. Larnder, Amee R. Manges, Rachel A. Murphy

**Affiliations:** ^1^ School of Population and Public Health University of British Columbia Vancouver Canada; ^2^ British Columbia Centre for Disease Control Vancouver Canada; ^3^ Cancer Control Research BC Cancer Vancouver Canada

**Keywords:** breast cancer, estrobolome, estrogen, gut microbiome, metagenomics

## Abstract

Increasing evidence links the gut microbiome to carcinogenesis. Disruptions in estrogen regulation by the estrobolome—gut microbiota with estrogen‐related functions—may promote breast cancer. However, precise information on estrobolome targets and their underlying mechanisms is limited. This review identifies relevant targets for measuring the estrobolome, focusing on enzymes and microbial taxa involved in processing estrogens, precursors, metabolites, and phytoestrogens, to facilitate the exploration of potential links to breast cancer. Evidence from breast cancer case–control studies is synthesized to assess alignment with these targets, highlight gaps in the evidence, and suggest new paths forward. Findings from case–control studies were heterogeneous and showed limited alignment with estrobolome targets, with only *Escherichia coli* and *Roseburia inulinivorans* identified as differentially abundant and functionally relevant between cases and controls. The lack of compelling evidence for estrobolome‐specific mechanisms may reflect measurement challenges or may suggest that broader ecological changes in the microbiome, which influence a network of interacting mechanisms, are more influential for carcinogenesis. To clarify the estrobolome's role in breast cancer, future research should use advanced sequencing techniques and methods such as metabolomics and transcriptomics, while considering clinical and behavioral factors that may modify estrobolome mechanisms.

## Graphical abstract

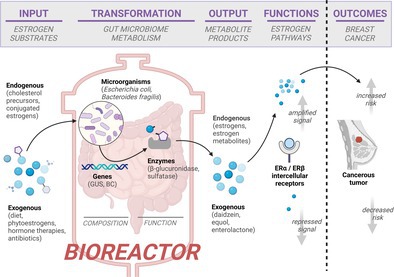

**Abbreviations**
ATCC: American Type Culture Collection
DCNC: Dunn Clinical Nutrition Centre
DHEA: dehydroepiandrosterone
DSM: Deutsche Sammlung von Mikroorganismen und Zellkulturen
EC: enzyme commission
EM: estrogen metabolites
ER: estrogen receptor
HR+: hormone‐receptor positive
HSD: hydroxysteroid dehydrogenase
IgA: immunoglobulin A
JCM: Japan Collection of Microorganisms
LEfSe: linear discriminant analysis coupled with effect size
NCBI: National Centre for Biotechnology Information
NCFB: National Collection of Food Bacteria
NCTC: National Collection of Type Cultures
OH: hydroxyl group
SECO: secoisolariciresinol

## 1 INTRODUCTION

### 1.1 Breast cancer overview

Breast cancer incidence has risen over the past two decades.^1^, ^2^ Hormone receptor positive (HR+) breast cancer is the most prevalent subtype, accounting for around 75% of cases,^3^ in which elevated estrogen levels are critical to pathogenesis.^4^, ^5^, ^6^, ^7^ Most breast cancers occur in women aged 50 and older,^8^ and high systemic estrogen levels have been shown to contribute to HR+ breast cancer in postmenopausal women.^5^, ^9^, ^10^ Risk factors associated with increased estrogen exposure include early menarche, late menopause, and older age at first pregnancy, while other factors such as obesity, physical inactivity, and diet may also contribute to breast cancer indirectly through hormonal or metabolic pathways.^5^, ^11^, ^12^ While some estrogen‐related mechanisms, such as the promotion of cell proliferation and inhibition of apoptosis,^13^, ^14^ are well understood, the interplay between systemic estrogen levels and other factors, including the gut microbiome, remains less explored. A better understanding of these pathways is needed as current prevention strategies, which focus on general behaviour changes (e.g., increased physical activity), have demonstrated limited success in reducing the population burden of breast cancer.^12^, ^15^

### 1.2 The gut microbiome and its implications for breast cancer

**FIGURE 1:**
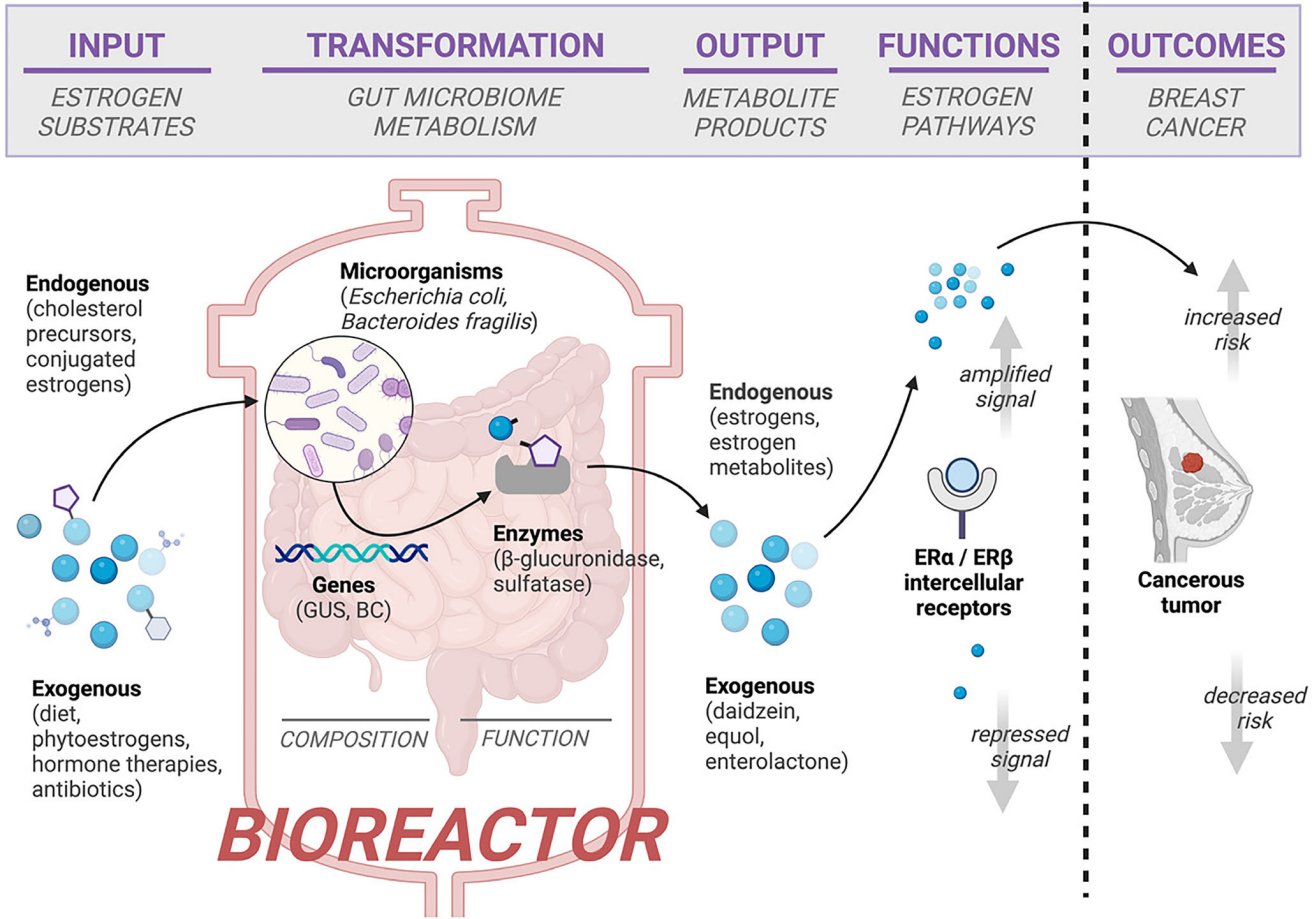
The gut microbiome can be conceived as a bioreactor, taking biochemical inputs (e.g., conjugated estrogens and phytoestrogens) and transforming them into estrogen forms that may influence breast cancer risk. External factors, such as nutrition, physical activity and obesity, can alter microbiome composition and potentially shift functions towards greater estrogen recycling, increasing breast cancer risk. BG, novel β‐glucuronidase gene; ER, estrogen receptor; GUS, widespread β‐glucuronidase gene. Created in https://BioRender.com.

The gut microbiome can be conceptualized as a bioreactor (Figure 1), a complex environment containing trillions of microorganisms with genes encoding enzymes for specific metabolic functions. These microorganisms are involved in various *transformations* of metabolic *inputs* into *outputs* within the intestinal tract, such as carbohydrate fermentation and amino acid metabolism.^15^ The resulting outputs may then influence numerous proximal and distal *functions* in the body, such as nutrient absorption, colonization resistance to pathogens, and systemic inflammation,^15^, ^16^, ^17^, ^18^ which can affect health *outcomes* (Figure 1). These diverse microbiome‐modulated functions operate within a dynamic equilibrium that is crucial for maintaining overall health and homeostasis^19^; however, deviations from this balance, such as decreased microbial diversity due to antibiotic use, can disrupt functions and potentially contribute to disease states.^16^, ^20^, ^21^, ^22^

Research has shown that the gut microbiome can modulate estrogen levels in the body via microorganisms carrying estrogen‐related functional genes, collectively known as the “estrobolome.”^19^ This has prompted exploration into the role of the estrobolome in the etiology of breast cancer.^5^ A key estrobolome mechanism is through the function of microbial β‐glucuronidases, where circulating estrogens are conjugated in the liver—reducing their reactivity—and then excreted into bile where, through enterohepatic circulation, they reach the small intestine.^5^, ^23^, ^24^, ^25^ Rather than being excreted, β‐glucuronidases deconjugate (*transformation*) the conjugated estrogens (*input*) into parent estrogens (*output*) that can be reabsorbed into circulation and interact with distal estrogen receptors α and β (*functions*) in breast tissue, thereby modulating overall breast cancer risk (*outcome*) (Figure 1).

While the estrobolome hypothesis is compelling, no specific microbial profile has been linked to breast cancer. Research has observed lower microbial diversity and higher proportions of facultative aerobes in breast cancer cases,^16^, ^26^, ^27^ suggesting broad ecological shifts in the microbiome. Although estrogens play a key role, most cancers are heterogeneous and involve a complex interplay of genetic, cellular, and environmental mechanisms, some of which may be influenced by the microbiome.^17^, ^28^, ^29^ Common behavioral factors like nutrition and physical activity—known breast cancer risk factors—also shape the broader microbiome, potentially influencing processes like inflammation and immune modulation linked to cancer development.^30^, ^31^ It therefore remains unclear whether specific estrogen‐related mechanisms are sufficient to drive carcinogenesis or if broader microbiome shifts play a more significant role, highlighting the need for further evidence.

### 1.3 Literature gaps in estrobolome research

Gaps remain in understanding the mechanisms by which the estrobolome contributes to carcinogenesis. In vitro and in vivo studies have established the plausibility of estrobolome mechanisms by identifying microbial enzymes capable of metabolizing conjugated estrogens and estrogen‐related compounds along with associated microbial taxa.^32^, ^33^, ^34^, ^35^ However, only a few mechanistic studies have been published, and they have a narrow focus on β‐glucuronidases, limiting evidence beyond this enzyme class. Understanding a broader range of estrogen‐related pathways is necessary to better define the estrobolome and identify specific targets. This, in turn, will help explore the estrobolome's role in breast cancer.

While certain gut microorganisms may carry genes for estrogen‐related enzymes, it remains unclear whether these genes are actively expressed and if the enzyme function observed in animal models is preserved in humans. Research on the estrobolome in humans is observational, limited to a small number of molecular epidemiological studies that compare differentially abundant microbial taxa between breast cancer cases and controls.^26^, ^27^, ^36^ Much of the literature consists of reviews,^11^, ^15^, ^22^, ^25^, ^37^, ^38^, ^39^, ^40^, ^41^ which fail to synthesize the limited primary research or assess the consistency of findings across studies, thus not addressing variability in study design and measurement limitations. Additionally, the focus often remains on microbial composition (Figure 1), which limits our understanding of the estrobolome's functional roles in breast cancer development. As a result, identifying the next steps to advance our understanding of the gut microbiome and its relationship to breast cancer remains challenging.

This review conceptualizes the gut microbiome as a bioreactor (Figure 1), highlighting its dynamic roles in estrogen regulation and potential implications for breast cancer. It is the first to synthesize primary evidence from in vitro, in vivo, and human studies to identify relevant targets for measuring the estrobolome, thereby facilitating the exploration of potential links to breast cancer and defining targets for future studies. This review aims to broaden the scope of the estrobolome beyond enzymes and microbial taxa directly related to estrogens, including those involved in metabolizing estrogen precursors and metabolites, as these may indirectly influence estrogen levels and, consequently, breast cancer risk. In light of the observational nature of molecular epidemiology studies, the bioreactor framework is also used to connect different types of evidence, exploring whether the epidemiologic findings comparing breast cancer cases to controls align with the estrobolome targets identified in mechanistic and animal model studies.

The first objective identified and summarized biochemistry reviews detailing estrogen‐related reactions in the human body, along with in vitro and in vivo studies demonstrating enzymes and microbial taxa with active estrogen‐related functions. This allowed the compilation of a comprehensive list of relevant metabolites, microbial enzymes, and microbial taxa as estrobolome targets. To clearly define these targets and facilitate better standardization for practical applications of our findings, relevant biochemical reactions were linked to pathways outlined in the MetaCyc database (SRI International, CA, USA). Individual enzymes and linked enzyme commission (EC) numbers were defined using the Kyoto Encyclopedia of Genes and Genomes (Kanehisa Laboratories, Japan). Additionally, to address reclassification of microbial taxonomy over time, Taxonomy IDs from the National Centre for Biotechnology Information (NCBI) (National Institutes of Health, USA) were included.

The second objective summarized molecular epidemiologic studies and investigated whether microbial taxa differentially abundant in breast cancer cases or associated with estrogen‐related compounds aligned with estrobolome targets identified in the first objective. Studies examining taxonomic relationships to breast cancer or estrogens were identified by searching biological databases using terms “gastrointestinal microbiome” and “breast neoplasms or breast carcinoma in situ or carcinoma, ductal, breast,” or “estrogens, estradiol, estrogen metabolites, estrogens catechol” and by searching article bibliographies. These included case–control or cross‐sectional studies of biological females with a proportion of postmenopausal women (with hormone‐receptor positive cancer for case–controls) among the research subjects. Methods included 16S rRNA or whole metagenome sequencing of human stool collected prior to any cancer‐related therapies to assess the gut microbiota, while reporting taxa differentially abundant between breast cancer cases and controls or significantly correlated to estrogens.

## 2 MECHANISTIC AND ANIMAL MODEL STUDIES: IDENTIFYING ESTROBOLOME TARGETS

### 2.1 Key estrogen‐related metabolites and reactions

Estrogens are steroid hormones derived from cholesterol precursors, with estradiol being the most potent form, followed by estrone.^9^, ^25^ They can be metabolized into various forms, including catechol estrogens through irreversible hydroxylations and estrogen conjugates via reversible conjugations, some of which have reduced reactivity or are biologically inactive.^42^ A higher ratio of metabolites to parent estrogens has been associated with decreased breast cancer risk.^37^ However, certain estrogen metabolites, such as 4‐hydroxy and 16‐hydroxy catechols, exhibit carcinogenic potential,^30^, ^31^ highlighting the need to consider metabolic pathways beyond parent estrogens in breast cancer. As changes in cholesterol precursors can indirectly influence estrogens, and metabolism can generate estrogen metabolites that may be carcinogenic or biologically inactive, both precursors and metabolites represent relevant groups for estrobolome targets.

When considering endogenous estrogens targets, it is important to consider menopausal status due to hormonal differences before, during, and after menopause. At menopause, estradiol production in the ovaries ceases, reducing systemic estrogens to similar levels as those in males.^9^, ^39^ Estrone becomes the primary estrogen, produced via peripheral conversion of androgens by aromatases in adipose tissue, which helps explain why obesity is a stronger breast cancer risk factor post menopause.^39^ Amid the strong estrogen fluctuations over ages when menstruation is occurring, microbial enzymes are less likely to significantly alter systemic estrogen levels; however, after menopause when estrogens circulate stably at lower levels, estrobolome perturbations may become more influential to breast cancer risk. Therefore, estrobolome mechanisms may be particularly relevant to estrone and androgens within a postmenopausal population.

Another group of relevant metabolites includes phytoestrogens—plant compounds structurally similar to estrogens that are found in a variety of foods, such as soybeans, flaxseeds, and berries.^43^ Key phytoestrogen classes include isoflavones (e.g., daidzein, genistein, equol^39^) and lignans (enterodiol and enterolactone^44^). During digestion, the microbiome can transform inactive phytoestrogen glycosides from foods into metabolically active aglycones that can enter circulation and bind estrogen receptors α and β (Figure 1).^39^ While phytoestrogens have a lower binding affinity than estrogens, they can circulate at higher concentrations and compete for binding sites, potentially exerting anti‐estrogenic effects.^39^ This may explain the association between high levels of phytoestrogens and reduced breast cancer risk.^45^ Similar to estrogens, circulating phytoestrogen aglycones can be conjugated in the liver and later deconjugated by the microbiome.^39^, ^46^

### 2.2 Establishing targets within the estrobolome

Microbiome bioreactor functions (Figure 1) were explored beyond the direct metabolism of estrogens to consider precursors, metabolites, and phytoestrogens. Four classes of microbial enzymes involved in estrogen‐related metabolism were identified from the literature. Table 1 outlines enzymes and EC numbers, hypothesized biochemical reactions, and corresponding reference pathways. Seventeen primarily in vitro studies (except for one genome database study^37^) were identified that functionally link microbial taxa to these enzymes, with a summary of findings and corresponding NCBI Taxonomy IDs provided in Table 2.

**TABLE 1:** Summary of evidence from biochemistry reviews and in vitro work mapping microbial enzymes to the metabolic transformations of estrogens and estrogen‐like compounds.

Microbial enzyme	Relevant metabolic reactions	Relevant pathways (MetaCyc)
**β‐Glucuronidases** (EC 3.2.1.31)	**Cholesterol precursors** *Plausible deconjugation of precursor glucuronides* **Estrogens** Estradiol‐3‐glucuronide ⟶ estradiol^38^ Estradiol‐17‐glucuronide ⟶ estradiol^33^, ^38^ Estrone‐3‐glucuronide ⟶ estrone^33^ **Estrogen metabolites** 4OH‐estradiol 4‐glucuronide ⟶ 4OH‐estradiol^38^ 4OH‐estradiol‐3‐glucuronide ⟶ 4OH‐estradiol^38^ 16OH‐estrone 3‐glucuronide ⟶ 16OH‐estrone^38^ 16OH‐estrone 16‐glucuronide ⟶ 16OH‐estrone^38^ *Plausible deconjugation of other catechol glucuronides* **Phytoestrogens** Genistein β‐glucuronide ⟶ genistein^47^ Daidzein glucuronide ⟶ daidzein^48^ Enterodiol glucuronide ⟶ enterodiol^49^, ^50^	GLUCUOCAT‐PWY (superpathway of β‐D‐glucuronosides degradation) PWY‐7427 (β‐D‐glucuronide and D‐glucuronate degradation)
**Sulfatases** Steroid sulfatase (EC 3.1.6.2) **Sulfotransferases** Steroid sulfotransferase (EC 2.8.2.15) Alcohol sulfotransferase (EC 2.8.2.2) Aryl sulfotransferase (EC 2.8.2.1) Estrone sulfotransferase (EC 2.8.2.4)	**Cholesterol precursors** DHEA‐sulfate ⟶ DHEA^51^, ^52^ *Plausible deconjugation of precursor sulfates* **Estrogens** Estrone‐3‐sulfate ⟶ estrone^34^, ^52^, ^53^ Estradiol‐3‐sulfate ⟶ estradiol^53^ **Estrogen metabolites** *Plausible deconjugation of catechol estrogen sulfates* **Phytoestrogens** Daidzein‐sulfate ⟶ daidzein^48^ Genistein‐sulfate ⟶ genistein^48^	
**3β‐HSDs** 3β‐hydroxy‐Δ5‐steroid dehydrogenase (EC 1.1.1.145) 3β‐hydroxysteroid 3‐dehydrogenase (EC 1.1.1.270)	**Cholesterol precursors** Pregnenolone ⟶ progesterone^52^, ^54^ 17OH‐pregnenolone ⟶ 17OH‐progesterone^54^, ^55^ DHEA ⟶ androstenedione^52^, ^54^, ^55^ Androstenediol ⟶ testosterone^55^	PWY‐8151 (cholesterol degradation to androstenedione III [anaerobic]) PWY‐8152 (androstenedione degradation II [anaerobic]) PWY‐8155 (testosterone degradation [anaerobic]) PWY‐6943 (testosterone and androsterone degradation to androst‐4‐en‐3,17‐dione) PWY‐7305 (superpathway of steroid hormone biosynthesis) PWY‐7299 (progesterone biosynthesis) PWY66‐377 (pregnenolone biosynthesis) PWY66‐378 (androgen biosynthesis) PWY66‐380 (estrogen biosynthesis I) PWY‐7306 (estradiol biosynthesis II)
**17β‐HSDs** 3(or 17)‐β‐hydroxysteroid dehydrogenase (EC 1.1.1.51) 17β‐estradiol 17‐dehydrogenase (EC 1.1.1.62) 3α‐(17β)‐hydroxysteroid dehydrogenase (NAD[+]) (EC 1.1.1.239) Testosterone 17‐beta‐dehydrogenase (NADP[+]) (EC 1.1.1.64)	**Cholesterol precursors** DHEA ${⇌}_{17\beta 2}^{17\beta 1}$ androstenediol^9^, ^52^, ^55^ Androstenedione ${⇌}_{17\beta 2}^{17\beta 3,5}$ testosterone^9^, ^52^, ^55^ **Estrogens** Estrone ${⇌}_{17\beta 2}^{17\beta 1}$ estradiol^9^, ^52^, ^55^ Estriol ${⇌}_{17\beta 1}^{17\beta 2}$ 16OH‐estrone^51^	
**β‐Glucosidases** (EC 3.2.1.21)	**Phytoestrogens** Genistin ⟶ genistein^39^, ^56^ Daidzin ⟶ daidzein^39^, ^56^ Glycitin ⟶ glycitein^39^ SECO diglucoside ⟶ SECO^57^ Pinoresinol diglucoside ⟶ pinoresinol^57^	PWY‐2002 (isoflavonoid biosynthesis I) PWY‐2083 (isoflavonoid biosynthesis II)

*Note*: Plausible was noted for substrates when there was a biochemical basis but no current evidence available for their function with the microbial enzyme. Individual enzymes and linked enzyme commission (EC) numbers were identified using the Kyoto Encyclopedia of Genes and Genomes (Kanehisa Laboratories, Japan). Relevant pathways were selected from the MetaCyc Metabolic Pathway Database (SRI International, CA, USA).

Abbreviations: DHEA, dehydroepiandrosterone; EC, enzyme commission number; HSD, hydroxysteroid dehydrogenase; PWY, pathway; SECO, secoisolariciresinol.

**TABLE 2:** Summary of in vitro evidence for bacteria found in the human gut microbiota capable of enzyme functions that participate in the metabolism of estrogens and related compounds.

Taxonomy	β‐Glucuronidases	Sulfatases + Sulfotransferases	3β‐HSDs	17β‐HSDs	β‐Glucosidases
**Actinobacteria**					
Bifidobacterium (1678)	37				
*Bifidobacterium adolescentis (1680)—DSM 20083 (367928), L2‐32*	58				35, 58
*Bifidobacterium angulatum (1683)—DSM 20098 (518635), NCFB 2237*	59				35, 59
*Bifidobacterium bifidum (1681)—NCFB 2203, NCFB 2454*	59				59
*Bifidobacterium breve (1685)—DSM 20213 (518634)*	58, 59				35, 58, 59
*Bifidobacterium pseudocatenulatum (28026)—DSM 20438 (547043)*					35
*Bifidobacterium pseudolongum (1694)—NCFB 2244 (1437595)*	59				59
*Bifidobacterium dentium (1694)*	32				
*Bifidobacterium longum (1963)—JCM 1217 (565042), NCFB 2716*	58, 59				58, 59
Collinsella (102106)	37				
*Collinsella massiliensis (1232426)*		34			
*Collinsella aerofaciens (74426)—JCM 7790*	58				58
*Eggerthella lenta (84112)*			60, 61	60	
*Eggerthella* sp. *CAG:298 (1262876)*			62		
Dermabacter (36739)	37				
Propionibacterium (1743)	37				
**Proteobacteria**					
Citrobacter (544)	37				
Escherichia (561)	37				
*Escherichia coli (562)—DH10B (316385), L95, K12 (83333), DCNC 20*	32, 33, 59, 63	34			59
Edwardsiella (635)	37				
Tannerella (195950)	37				
**Bacteroidetes**					
Bacteroides (816)	37				
*Bacteroides distasonis (823)*	58				58
*Bacteroides fragilis (817)—NCFB 2217*	33, 58, 59	34	61	64	58, 59
*Bacteroides capillosus (106588)—ATCC 29799 (411467)*	32				
*Bacteroides thetaiotaomicron (818)—DSM 2079 (226186)*	58	65, ^a^			35, 58
*Bacteroides ovatus (28116)—DCNC 11, ATCC 8483 (411476)*	32, 59				59
*Bacteroides uniformis (820)—JCM 5828 (411479)*	58				58
*Bacteroides vulgatus (39485)—DCNC 23, JCM 5826 (435590)*	58, 59				58, 59
Alistipes (239759)	37				
*Alistipes obesi (2585118)*		34			
*Parabacteroides johnsonii (387661)—DSM 18315 (537006)*	32				
*Parabacteroides merdea (46503)—ATCC 43184 (411477)*	32				
**Firmicutes**					
*Hungatella hathewayi (154046)*		34			
*Enterococcus faecalis (1351)—DCNC 24*	59				59
*Enterococcus faecium (1352)—DCNC 26*					59
*Eubacterium rectale (39491)—A1‐86 (657318), M104/1 (657317), T1‐815*					35
*Eubacterium siraeum (39492)—70/3 (657319)*					35
*Eubacterium eligens (39485)*	33				
*Butyrivibrio fibrisolvens (831)—16/4 (657324)*					35
Clostridium (1485)	37				
*Clostridium bartlettii (261299)—DSM 16795 (445973)*	32				
*Clostridium bifermentans (1490)—NCFB 2189*	59				59
*Clostridium butyricum (1492)—DCNC 19*	59				59
*Clostridium clostridioforme (1531)—JCM 1291*	58				58
*Clostridium innocuum (1522)*	58		66		58
*Clostridium paraputrificum (29363)—JCM 1293*	58				58
*Clostridium perfringens (1502)—JCM 1290 (195103), JCM 3817 (451754), NCTC 8679*	33, 58, 59				58, 59
*Clostridium ramosum (1547)*	58				58
*Clostridium septicum (1504)—NCFB 2189*					59
*Clostridium sporongenes (1509)—DCNC 5*					59
*Clostridium* sp. *Marseille‐P2538 (1816694)*	33				
*Coprococcus eutactus (33043)*					35
*Coprococcus* sp. *ART55/1 (751585)*					35
*Coprococcus* sp. *L2‐50*					35
Marvinbryantia (248744)	37				
*Marvinbryantia formatexigens (168384)—DSM 14469 (478749)*	32				
Roseburia (841)	37				
*Roseburia hominis (301301)—A2‐181, DSM 16839 (585394)*	33, 35				35
*Roseburia intestinalis (166486)—L1‐8151, DSM 14610 (536231), L1‐195, M50/1 (657315)*	35				35
*Roseburia inulinivorans (360807)—DSM 16841 (622312)*	32, 33				35
*Roseburia faecis (301302)—DSM 16840 (451638)*					35
Lactobacillus (1578)	37				
*Lactobacillus acidophilus (1579)—DCNC 1237*	59				59
*Lactobacillus gasseri (1596)*	32, 63				
*Lactobacillus rhamnosus (47715)*	33				
Ruminococcus	37				
*Ruminococcus gnavus (33038)—ATCC 29149 (411470)*	32, 33		67		
*Ruminococcus* sp. *80/3 (1160721)*					35
*Ruminococcus obeum (40520)—A2‐162 (657314)*					35
*Peptococcus niger (2741)*		53			
Faecalibacterium (216851)	37				
*Faecalibacterium prausnitzii (853)—DSM 17677 (411483), L2‐6 (718252), M21/2 (411485)*	32, 33, 35				
*Streptococcus agalactiae (1311)*	33				
*Subdoligranulum variabile (214851)—DSM 15176 (411471)*	32				

*Note*: Taxonomy is described where bold text refers to phylum level, regular text refers to genus level, *italicized text* refers to species level, and hyphens separate strain level. National Center for Biotechnology Information (NCBI) taxonomy IDs are included in parentheses for standardization in naming. All studies conducted in vitro work, except Kwa et al.^37^ indexed the Human Microbiome Project database to identify gut microbiota genomes associated with the presence of β‐glucuronidase—genera reported to have any positive β‐glucuronidase genes were included.

Abbreviations: ATCC, American Type Culture Collection; DCNC, Dunn Clinical Nutrition Centre; DSM, Deutsche Sammlung von Mikroorganismen und Zellkulturen; HSD, hydroxysteroid dehydrogenase; JCM, Japan Collection of Microorganisms; NCFB, National Collection of Food Bacteria; NCTC, National Collection of Type Cultures.

^a^ Specific to sulfotransferases, whereas all other citations in this enzyme class are specific to sulfatases.

#### 2.2.1 β‐Glucuronidases

β‐Glucuronidases are solely produced by microbes and deconjugate estrogen glucuronides into active estrogens.^33^, ^38^, ^68^ While estrobolome studies have focused on this mechanism, their functional roles extend further (Table 1). A number of catechol estrogens can undergo glucuronidation,^9^, ^68^ with β‐glucuronidase action reported on the 4‐ and 16‐hydroxy glucuronide metabolites.^38^ Among phytoestrogens, β‐glucuronidase action has been reported for both isoflavone and enterolignan glucuronides,^39^, ^46^ including genistein‐, daidzein‐, and enterodiol‐glucuronides into their active aglycone forms.^47^, ^48^, ^49^, ^50^ Many cholesterol precursors exist as glucuronides, such as androsterone and androstenediol glucuronides,^69^ dehydroepiandrosterone (DHEA) 3‐glucuronide, and pregnanediol 3‐glucuronide^70^; however, these metabolites have not yet been characterized as substrates for β‐glucuronidase enzymes.

Table 2 lists taxa associated with β‐glucuronidase activity, spanning 20 different genera. Among these, the genera from the Actinobacteria (*n* = 4) and Firmicutes (*n* = 9) phyla have the highest representation of β‐glucuronidase activity. Several genera include multiple species capable of enzyme function, such as *Clostridium* spp. (*n* = 9), *Bifidobacterium* spp. (*n* = 7), *Bacteroides* spp. (*n* = 7), *Roseburia* spp. (*n* = 3), and *Lactobacillus* spp. (*n* = 3). In many cases, multiple strains within a given species were found to contain β‐glucuronidase activity, such as with *Clostridium perfringens JCM 1290*, *JCM 3817*, and *NCTC 8679*.^58^, ^59^

#### 2.2.2 Sulfatase and sulfotransferases

Sulfatase and sulfotransferases catalyze opposing reactions: sulfatases deconjugate sulfate groups to form active compounds, whereas sulfotransferases conjugate to deactivate compounds (Table 1).^51^ Most evidence pertains to their action on estrone 3‐ and estradiol 3‐sulfate.^34^, ^52^, ^53^ Both catechol estrogens^9^ and cholesterol precursors, such as DHEA‐sulfate,^51^, ^52^ can be sulfated, but there is limited research into microbial action on these substrates. Among phytoestrogens, sulfotransferases have been reported to conjugate genistein and daidzein into sulfate esters, along with their deconjugation by sulfatases.^39^, ^46^, ^48^

Two in vitro studies reported six species demonstrating sulfatase activity (Table 2): *Collinsella massiliensis*, *Escherichia coli*, *Bacteroides fragilis*, *Alistipes obesi*, *Hungatella hathewayi*,^34^ and *Peptococcus niger*.^53^ For sulfotransferase, one in vitro study reported *Bacteroides thetaiotaomicron*.^71^

#### 2.2.3 Hydroxysteroid dehydrogenases (HSDs)

HSDs have diverse roles in cholesterol processing, with 3β‐ and 17β‐HSD classes involved in estrogen‐related pathways (Table 1).^72^ 3β‐HSDs primarily act on progesterone and androgen precursors, including pregnenolone, 17OH‐pregnenolone, DHEA, and androstenediol.^52^, ^54^, ^55^ Many 17β‐HSD variants exist which facilitate interconversions of androgen precursors and estrogens, including DHEA to androstenediol, androstenedione to testosterone,^9^, ^52^, ^55^ estrone to estradiol,^9^, ^52^, ^55^ and estriol to 16OH‐estrone.^51^

Five species were found to demonstrate 3β‐HSD activity (Table 2): *Eggerthella lenta*,^60^, ^61^ *Eggerthella* sp. *CAG:289*,^62^ *Bacteroides fragilis*,^61^ *Clostridium innocuum*, and *Ruminococcus gnavus*.^67^ Two of these species, *Eggerthella lenta* ^60^ and *Bacteroides fragilis*,^64^ were also reported to have 17β‐HSD activity.

#### 2.2.4 β‐Glucosidases

β‐Glucosidases main estrobolome functions involve catalyzing the initial transformation of phytoestrogen glycosides from plant material into biologically active aglycones (Table 1).^39^, ^56^ At high concentrations, these aglycone forms can compete with estrogens for binding to ERα and ERβ in breast tissue, potentially exerting anti‐estrogenic effects. Among isoflavones, this is documented for daidzein and genistein.^39^, ^56^ For enterolignans, β‐glucosidases are involved at various steps in the production of secoisolariciresinol (SECO) and pinoresinol.^57^ As phytoestrogens are linked to a reduced risk of breast cancer, β‐glucosidases are expected to have a protective effect, in contrast to other enzyme targets whose functions may elevate breast cancer risk (e.g., β‐glucuronidase).

β‐Glucosidase activity spanned 12 genera (Table 2), with *Clostridium* spp. (*n* = 9), *Bifidobacterium* spp. (*n* = 7), *Bacteroides* spp. (*n* = 6), and *Roseburia* spp. (*n* = 4) having the highest representation of activity. Notably, a total of 26 species across eight genera demonstrated shared activity between both β‐glucosidase and β‐glucuronidase enzymes (e.g., *Clostridium perfringens, Roseburia inulinivorans*).

### 2.3 Limitations and future directions

We identified four microbial enzyme classes and 13 specific enzymes that may contribute mechanistic links between the gut microbiome and breast cancer through their metabolism of estrogen‐related compounds. To date, most research has focused on the activity of β‐glucuronidase and sulfatase on estrogens,^28^, ^29^ with limited understanding into their effects on estrogen precursors, metabolites, and phytoestrogens which may indirectly influence estrogen levels. More evidence is needed to confirm the specific functions of enzyme targets. Future studies should focus on validating new estrobolome targets through homology searches in large sequencing databases to identify taxa capable of these functions, followed by targeted in vitro and in vivo characterization on these precursor, metabolite, and phytoestrogen compounds.

Functional redundancy is a common feature of the gut microbiome community. As apparent in Table 2, multiple species can perform the same function (e.g., 20 genera capable of β‐glucuronidase activity) and some taxa share multiple pathways (e.g., *Bacteroides fragilis* can carry all four enzyme classes). This complicates the picture of estrobolome mechanisms, as β‐glucuronidase can deconjugate both endogenous estrogens and phytoestrogens, potentially resulting in either higher or lower estrogen levels. Functional redundancy also highlights that measuring microbiota composition may not be sufficient to detect important estrobolome functions. Shifting the focus to functional genes, pathways, or the direct measurement of metabolites and mRNA transcripts could better identify results that are causally related to breast cancer.

## 3 MOLECULAR EPIDEMIOLOGY STUDIES: CONNECTING TO ESTROBOLOME TARGETS

### 3.1 Summary of molecular epidemiology studies

#### 3.1.1 Gut microbiome and breast cancer

To explore evidence in humans, we reviewed nine molecular epidemiologic studies that examined the relationship between gut microbiota and breast cancer, focusing on studies that reported differentially enriched and depleted taxa in breast cancer cases relative to controls (Table 3).

**TABLE 3:** Summary of molecular epidemiologic studies examined for differentially abundant taxa between breast cancer cases and controls (*n* = 9) and associations of the gut microbiome to estrogen (*n* = 6).

Study	Experimental methods	Population	Findings (differentially abundant taxa between cases and controls/estrogen associations)
Exploring the association of gut microbiome to breast cancer (*n* = 9)			
Goedert *et al*.^81^ 2015^a^	16S rRNA V3–V4 sequencing	Sample of 96 postmenopausal women aged 50–74 years living in the United States. Included cases (*n* = 48) with any breast cancer type (87.5% ER+) and healthy controls (*n* = 48), where cases were treatment naïve.	Wilcoxon rank‐sum tests identified 8 differential genera, three enriched in breast cancer cases (*Dorea, Enterobacter*, *Ruminococcus*) and five in controls (e.g., *Alistipes, Marvinbryantia, Faecalibacterium*).
Zhu *et al*.^79^ 2018^b^	Metagenomic shotgun sequencing	Sample of 90 postmenopausal women^1^ living in China. Included cases with any breast cancer type (*n* = 44) and healthy controls (*n* = 46), where cases were treatment naïve.	Wilcoxon rank‐sum tests identified 22 differential species (across 12 genera), 19 enriched in cases (e.g., *Escherichia coli, Prevotella amnii, Enterococcus gallinarum, Lactobacillus mucosae*) and three in controls (e.g., *Roseburia inulinovorans*).
Byrd *et al*.^26^ 2021	16S V4 rRNA sequencing	Sample of 895 pre and postmenopausal women aged 18–74 years living in Ghana. Included cases (*n* = 379) with any breast cancer type (31.4% triple negative), non‐malignant tumor controls (*n* = 102), and healthy controls (*n* = 414), where cases were treatment naïve.	Unconditional multivariable polytomous logistic regression adjusted for nine variables identified 18 differential genera, six enriched in cases (*Bacteroides, Enterorhabdus, Flavinofractor, Oxalobacter, Oscillibacter, Ruminococcus*) and 12 in controls (e.g., *Romboutsia, Faecalibacterium, Bifidobacteria, Roseburia*).
Bobin‐Dubigeon *et al*.^75^ 2021	16S rRNA V3‐V4 sequencing	Sample of 55 pre and postmenopausal women living in France. Included cases (*n* = 25) with any breast cancer type (100% ER/Pg+) and healthy controls (*n* = 30), where cases were treatment naïve.	ANOVA adjusted for age identified four differential genera, one enriched in cases (*Lachnospira*) and three in controls (*Butyricimonas, Coprococcus, Odoribacter*).
Yang *et al*.^76^ 2021	16S rRNA V4 sequencing	Sample of 102 pre and postmenopausal women living in China. Included cases (*n* = 83) with any breast cancer type (61% ER+) and non‐malignant tumor controls (*n* = 19), where cases were treatment naïve.	LEfSe with LDA > 2 identified 24 differential genera, one enriched in cases (*Citrobacter*) and 23 in controls (e.g., Butyrivibrio, Clostridium, Romboutsia, Faecalibacterium).
Ma *et al*.^27^ 2022	16S rRNA V3‐V4 sequencing	Sample of 46 pre and postmenopausal women living in China. Included cases with any breast cancer type (*n* = 26) and healthy controls (*n* = 20), where cases were treatment naïve.	LEfSe with LDA > 2 identified 12 differential genera, four in cases (*Escherichia, Lactobacillus, Porphyromonas, Peptoniphilus*) and eight in controls (e.g., *Anaerofilum, Butyricimonas, Faecalibacterium*).
Kwa *et al*.^77^ 2022^c^	16S rRNA V4 sequencing	Sample of 68 postmenopausal women living in the United States. Included cases with ER and/or Pg+ and HER2– breast cancer (*n* = 46) and healthy controls (*n* = 22), where cases were prior to adjuvant endocrine therapy.^c^	LEfSe with LDA > 2 identified three differential genera, all enriched in controls (*Coprococcus, Ruminococcus, Holdemania*). Four differential species were reported, one enriched in cases (*Bifidobacterium animalis*) and three in controls (*Coprococcus catus, Butyricicoccus pullicaecorum, Ruminococcus lactaris*).
Altinok Dindar *et al*.^74^ 2023	16S rRNA V4 sequencing	Sample of 86 women aged 20–89 years living in the United States. Included cases (*n* = 42) with any breast cancer type (86% ER+) and healthy controls (*n* = 44), where cases were treatment naïve.	LEfSe with LDA > 2 identified six differential genera, three enriched in cases (*Acidaminococcus, Hungatella, Tyzzerella*) and three in controls (*Anaerofilum, Dialister, Romboutsia*).
Su *et al*.^78^ 2024	16S rRNA V3‐V4 sequencing	Sample of 82 pre and postmenopausal women living in China. Included cases with any breast cancer type (n=42) and benign breast disease controls (n=40), where cases were treatment naïve.	LEfSe with LDA > 2 identified 10 differential genera, four enriched in cases (*Prevotella, Shigella, Succinispira, Selenomonas*) and six in controls (e.g., *Bifidobacterium, Ruminococcus, Faecalibacterium*). One species was reported to be enriched in cases (*Prevotella copri*)
Exploring the association of gut microbiome to estrogens (*n* = 6)			
Yaghjyan *et al*.^24^ 2023	Cross‐sectional 16S rRNA V1‐V2 sequencing (stool) LC‐high resolution MS (urine)	Sample of 164 postmenopausal women living in the United States. Metabolites: Parent estrogens (estrone, estradiol); 13 EMs (2‐hydroxyestrone, 2‐methoxyestrone, 2‐hydroxyestradiol, 2‐methoxyestradiol, 3‐methoxyestrone, 4‐hydroxylated EMs, 16‐hydroxylated EMs).^d^	Spearman correlations showed associations of EM ratios with alpha diversity measures, finding: positive association of 2‐catechols:methylated 2‐catechols with Shannon index; inverse association of E1:Total and 4‐EM:2‐EM, and positive association of 2‐EM:Parent with Chao1; inverse association of 4‐EM:Total, 4‐EM:Parent, 4‐EM:2‐EM, and 4‐EM:16:EM, and positive association of 2‐EM:Parent with Phylogenetic diversity.
Kwa *et al*.^77^ 2022^c^	Case‐control 16S rRNA V4 sequencing (stool) orbitrap LC‐MS (plasma, urine)	Sample of 68 postmenopausal women living in the United States. *See above*. Metabolites: Parent estrogens (estrone, estradiol); 9 EMs (2‐hydroxyestrone, 2hydroxyestradiol, 2‐methoxyestrone, 2‐hydroxy‐3‐Omethylestrone, 4‐hydroxyestrone, 4‐hydroxyestradiol, 16α‐hydroxyestrone, 16‐ketoestradiol, estriol); progesterone, testosterone	Sorting relative taxonomic abundance in samples by progesterone level showed that high progesterone levels may lead to altered microbial composition, with *Blautia* and *Ruminococcaceae* being possible relevant taxa.
Shin *et al*.^83^ 2019	Cross‐sectional 16S rRNA V1‐V2 sequencing (stool) Electrochemiluminescence immunoassay (serum)	Sample of 57 (31 male, 26 female) aged 25‐65 years living in Korea. Metabolites: Estradiol	Women with the highest estradiol levels had increased Bacteroidetes and decreased Firmicutes. Spearman's rank correlation coefficient showed the relative abundances of *Slackia* and *Butyricimonas* were significantly and negatively correlated with the levels of serum estradiol.
Goedert *et al*.^81^ 2015^a^	Case‐control 16S rRNA V3–V4 sequencing (stool) LC‐MS/MS (urine)	Sample of 96 postmenopausal women aged 50–74 years living in the United States. *See above*. Metabolites: Parent estrogens (estrone, estradiol); 13 EMs (2‐hydroxylated, 4‐hydroxylated, and 16‐hydroxylated).^d^	Estrogen associations with microbiota alpha diversity were tested by Spearman rank‐order correlation. Total estrogens correlated with α‐diversity in control patients (p 0.009) but not case patients (p 0.77).
Flores *et al*.^23^ 2015	Cross‐sectional 16S rRNA V1‐V2 sequencing (stool) LC‐MS/MS (stool, urine)	Sample of 51 (25 male, 26 female – 19 postmenopausal) living in the United States. Metabolites: Parent estrogens (estrone, estradiol); 13 EMs (2‐hydroxylated, 4‐hydroxylated, and 16‐hydroxylated).^d^	Pearson correlations showed urinary estrogens (and more EMs) were strongly associated with fecal microbiome alpha diversity, the order non‐Clostridiales, and three genera in the *Ruminococcaceae* family (*R. Oscillibacter, R. Subdoligranulum, R. genus NA*). Lower levels of fecal conjugated and deconjugated estrogens was associated with higher alpha diversity.
Fuhrman *et al*.^82^ 2014	Cross‐sectional 16S rRNA sequencing (stool) LC‐MS/MS (urine)	Sample of 60 postmenopausal healthy women living in the United States. Metabolites: Parent estrogens (estrone, estradiol); 13 EMs (2‐hydroxylated, 4‐hydroxylated, and 16‐hydroxylated).^d^	Multivariable linear regression evaluated metabolite associations with fecal microbiome diversity and taxonomy relative abundance, adjusting for age, BMI, enrollment, specimen temperature. Elevated EM:Parent was associated with alpha diversity, the order *Clostridiales*, and genus *Bacteroides*.

Abbreviations: ANOVA, analysis of variance; EM, estrogen metabolites; ER, estrogen receptor; HER2, human epidermal growth factor receptor 2; LC, liquid chromatography; LDA, linear discriminant analysis; LEfSe, linear discriminant analysis coupled with effect size; MS, mass spectrometry; Pg, progesterone.

^a^ The results were abstracted from the detailed supplemental materials for the article because the findings presented in the main text (which cite the supplemental materials) were inconsistent with the supplemental data.

^b^ Also included a subgroup of premenopausal women (18 cases, 25 controls) but results were not reported in this review.

^c^ This study is a pre‐print and not a peer‐reviewed publication. The definition of treatment‐naïve was not clear as they specified no prior adjuvant endocrine therapy nor antibiotic use in the past 6 months, but the timing of other cancer‐related therapies was not reported.

^d^ 2‐Hydroxylated EMs (2‐hydroxyestrone, 2‐methoxyestrone, 2‐hydroxyestradiol, 2‐methoxyestradiol, 2‐hydroxyestrone‐3‐methyl ether); 4‐hydroxylated EMs (4‐hydroxyestrone, 4‐methoxyestrone, 4‐methoxyestradiol); 16‐hydroxylated EMs (16α‐hydroxyestrone, estriol, 17‐epiestriol, 16‐ketoestradiol, 16‐epiestriol).

A total of 63 genera were differentially abundant between breast cancer cases and controls. There was considerable heterogeneity, with 45 (71%) genera reported in only one of the nine studies. Only 20 of the 63 total genera (31%) were reported as enriched in cases (Table 3), with 16 reported in only one of the nine studies and the remaining four contrastingly reported as depleted by a different study (*Acidaminococcus*,^27^, ^77^ *Dorea*,^26^, ^36^ *Lachnospira*,^26^, ^74^, ^75^ and *Ruminococcus* ^26^, ^36^, ^76^, ^78^). The remaining 43 of the 63 total genera were reported as depleted in cases, of which 31 were reported in only one study while eight were concordantly identified in two or more studies (*Faecalibacterium*,^26^, ^27^, ^36^, ^75^, ^78^ *Butyricimonas*,^27^, ^36^, ^74^ *Coprococcus*,^26^, ^74^, ^76^ *Romboutsia*,^26^, ^75^, ^77^ *Bifidobacterium*,^26^, ^78^ *Collinsella*,^32^, ^36^ *Anaerofilum*,^27^, ^77^ and *Epulopsicium* ^36^, ^78^). Across three studies,^73^, ^76^, ^78^ 26 species were reported as differentially abundant between cases and controls; however, each study reported a unique set of species.

Different sequencing methods and bioinformatics strategies led to results reported at different taxonomic levels (i.e., phylum versus genus or species level), making comparisons across studies challenging (Table 3). Eight of the nine studies employed 16S sequencing, which examines genetic variation in a single marker gene and is valid down to the genus—and not species—level. In contrast, one study^73^ utilized whole metagenome sequencing, which allows for deeper taxonomic resolution and provides more complete gene prediction and functional pathway annotation. All statistical analysis approaches offered limited adjustment for confounding variables, such as diet and physical activity (Figure 1).

Most studies (*n* = 7) had sample sizes smaller than 100, which, along with unbalanced case and control groups, such as 83 cases to 19 controls^75^ and 46 cases to 22 controls,^76^ limited statistical power (Table 3). The control selection strategy also differed, where six studies used a healthy control group and three used a control group with benign malignant tumors, which is itself a risk factor for breast cancer.^26^, ^75^, ^78^ Only one study focused exclusively on women with HR+ breast cancer,^76^ while the others included all breast cancer subtypes, some of which have aetiologies non‐specific to estrogen pathways, which could dilute estrogen‐specific associations. The selection criteria also differed, particularly regarding subject exclusions—or lack thereof (*n* = 2)^26^, ^74^—of gastrointestinal‐related illnesses or surgeries, which could significantly impact the gut microbiome.

#### 3.1.2 Gut microbiome and estrogens

To explore at another level of the bioreactor (Figure 1), we reviewed six epidemiologic studies examining the relationship between the gut microbiome and estrogens (Table 3). Two studies observed associations with overall alpha diversity,^24^, ^36^ while others identified seven specific genera: *Bacteroides* associated with the metabolite to parent estrogen ratio^80^; *Oscillibacter, Subdoligranulum*, and *Ruminococcaceae genus NA* with estrogens and metabolites^23^; *Slackia and Butyricimonas* with estradiol^79^; and *Blautia* with progesterone^76^ (Table 3).

While various microbiota‐estrogen associations were reported, many were at summary measures or high taxonomic levels, with the lowest resolution at the genus level. Most studies measured different combinations of estrogens and metabolites, with one study^79^ measuring only estradiol. Of the biological samples analyzed, one study measured blood serum^79^ while the remaining five measured urine; two studies additionally measured feces^23^ and blood plasma.^76^ These methodological differences may account for variations in associations, as circulating estrogens differ from those excreted after processing by the kidneys or gut microbiome.

### 3.2 Alignment with the estrobolome targets

As the estrobolome targets were identified in in vitro models, the functional relevance of these microbial enzymes to breast cancer risk is unclear. To gain insight into their potential relevance, we explored whether microbial taxa associated with breast cancer (including the direction: enriched or depleted) aligned with the microbial taxa associated with enzyme targets.

#### 3.2.1 β‐Glucuronidases

Given their key role in estrogen deconjugation, we might expect breast cancer cases to be enriched in taxa carrying these enzymes. Of the 20 genera showing β‐glucuronidase activity (Table 2), epidemiologic studies reported differential findings among 10 of these genera. As expected, *Bacteroides*,^26^ *Citrobacter*,^75^ *Lactobacillus*,^27^ *Escherichia*,^27^ and *Ruminococcus* ^26^, ^36^ were enriched in cases, although the abundance of *Ruminococcus* was also reported as depleted in cases in other studies.^76^, ^78^ In contrast, *Bifidobacterium*,^26^, ^78^ *Collinsella*,^26^, ^78^ *Clostridium*,^75^ *Marvinbryantia*,^36^ *Roseburia*,^26^ and *Faecalibacterium*,^26^, ^27^, ^36^, ^75^, ^78^ were depleted in cases. At the species level, *Escherichia coli* abundance was enriched in cases^73^ whereas *Roseburia inulinivorans* was depleted in cases.^73^

#### 3.2.2 Sulfatases and sulfotransferases

For sulfatases, we may expect enriched taxonomic abundance among cases due to the deconjugation of sulfated compounds. Aligning with the targets in Table 2, *Bacteroides*,^26^ *Hungatella*,^77^ and *E. coli* ^73^ were reported to be enriched in cancer cases, while *Alistipes* ^36^ and *Collinsella* ^32^, ^36^ were reported as depleted.

As sulfotransferases catalyze the reverse reactions, we may expect the opposite with depleted abundance of sulfotransferase‐carrying bacteria, yet we see enriched *Bacteroides* ^26^ in cancer cases, which contains the only reported sulfotransferase target carried by *B. thetaiotaomicron*.^65^

#### 3.2.3 Hydroxysteroid dehydrogenases (HSDs)

For 3β‐HSDs, we may expect enriched taxonomic abundance among cancer cases due to their roles converting cholesterol precursors in pathways that produce estrogens.^9^ While we identified *B. fragilis* as a species with 3β‐HSD carriage (Table 2), only an enriched abundance at the genus level (*Bacteroides*) was reported in cases.^26^ Similarly, for *C. innocuum* with 3β‐HSD carriage (Table 2), a depletion at the genus level (*Clostridium*) was reported in cancer cases.^75^

For 17β‐HSDs, an expected direction is less clear as this class contains many enzymes that catalyze the targeted reactions in both directions (Table 1). For the reported target of *B. fragilis*,^64^ we see enriched *Bacteroides* in cases.^26^

#### 3.2.4 β‐Glucosidases

Due to their function in processing phytoestrogen glycosides, we may expect a depletion of bacterial taxa among breast cancer cases. Of the 12 genera found to contain species with β‐glucosidase activity, the molecular epidemiologic studies reported findings for nine genera. As expected at the genus level, a depletion of *Bifidobacterium*,^26^, ^78^ *Butyrivibrio*,^75^ *Clostridium*,^75^ *Coprococcus*,^26^, ^74^, ^76^ *Roseburia*,^26^ and *Ruminococcus* ^76^, ^78^ was observed in cases, although *Ruminococcus* was also reported as enriched in cases in other studies.^26^, ^36^ *Lactobacillus*,^27^ *Bacteroides*,^26^ and *Escherichia* ^27^ were reported as enriched in cases. At the species level, a depletion in cases was reported for *R. inulinivorans* ^73^ (a species known to carry β‐glucosidase^35^) as expected, while an enrichment in cases was reported for *E. coli*.^73^

#### 3.2.5 Estrogen associations

Additionally, we explored whether the findings from epidemiologic studies exploring microbial taxa associated with estrogens aligned with the estrobolome targets. Of the seven identified genera, only two aligned with in vitro findings: *Bacteroides*, with seven species containing estrogen‐related functions, and *Subdoligranulum* containing the *Subdoligranulum variabile* target (Table 1).

### 3.3 Limitations and future directions

The presence of distinct microbiota profiles or specific enrichment/depletion of relevant microbial taxa across molecular epidemiology studies was inconsistent, raising questions about the reproducibility of results. While several differential genera were reported, the resolution is not specific enough to draw meaningful comparisons to the in vitro work, which is reported down to the species and strain level. Of the differential species reported, only two aligned with estrobolome targets: an enrichment of *Escherichia coli* in cases (linked to β‐glucuronidase activity) and a depletion of *Roseburia inulinivorans* (linked to β‐glucosidase activity). However, it remains unclear whether these alignments persist at the strain level, as most studies used 16S sequencing which limits taxonomic and functional resolution. In vitro studies indicate β‐glucuronidase activity in certain *E. coli* strains, such as *DH10B*, *L95*,^32^ *K12* ^63^ and *DCNC 20*,^59^ but not all strains may exhibit estrobolome functions. Furthermore, there may be potential inaccuracies in taxonomic annotation due to variability and ambiguities across different reference databases.

These limitations highlight the need for sequencing methods that provide greater taxonomic and functional resolution. Whole metagenome sequencing detects specific genes and pathways and is a step towards this goal, enabling more accurate insights into estrobolome function within the microbiome bioreactor. However, the presence and abundance of microbial taxa carrying genes for specific enzyme targets do not indicate whether they are functionally active—regulatory or transcriptional effects may influence gene expression. Future epidemiologic studies should combine metagenomic sequencing with other functional measurements, such as metatranscriptomics or metabolomics, which can be integrated through multi‐omics approaches.^81^

Small sample sizes and population heterogeneity, particularly in case and control selection, likely contribute to the inconsistent epidemiologic findings. Larger studies are needed that clearly report breast cancer subtype, as including non‐HR+ subtypes—where estrogen is not a primary driver—could weaken observed effects. Similarly, menopause status should be considered, as estrogen‐related microbial pathways may be more influential in postmenopausal women where estrogen levels are lower and more stable. Focusing on postmenopausal HR+ cases would provide a more relevant population for studying these pathways in breast cancer risk. Future studies could also explore the estrobolome hypothesis by expanding into other estrogen‐related cancers, such as endometrial cancer. There is also a notable lack of consideration for external factors that may partially explain the observed variability. Important clinical or behavioral factors, such as obesity,^11^, ^20^ low and high fiber diets,^15^, ^20^ and exercise^82^, ^83^—all recognized breast cancer risk factors—can shape the broader gut microbiome and potentially influence processes like inflammation and immune modulation. This may lead to interacting mechanisms that modify the relationship between the estrobolome and breast cancer. Epidemiologic studies need to appropriately adjust for these potentially modifying factors or stratify analyses. Moreover, lack of data on dietary intake in the case–control studies limits the exploration of phytoestrogen‐related mechanisms in the estrobolome.

The case–control study design has inherent limitations in exploring this relationship. While data was collected before the initiation of cancer‐related treatments in the included studies, it was obtained after breast cancer diagnosis, potentially reflecting lifestyle changes in response to diagnosis or breast cancer itself and associated metabolic changes. This raises the possibility of reverse causality, as it remains unclear whether observed microbiome changes contribute to breast cancer or result from the presence of disease or changes to behavior post‐diagnosis. Additionally, single time‐point sampling may not accurately capture true enrichment or depletion patterns, limiting the exposure assessment. Prospective studies could address these limitations, but few cohorts have stored stool samples with sufficiently long follow‐up periods for breast cancer identification. The high costs of stool collection, storage, and sequencing further limit the integration of metagenomic data into existing cohorts.

## 4 CONCLUSION

While current evidence does not appear to directly support an estrobolome‐specific mechanism that influences breast cancer risk, the focus on individual bioreactor components (Figure 1) in recent studies leads to an incomplete understanding of how the different components of the bioreactor may (or may not) contribute to carcinogenesis. The hypothesis that the estrobolome contributes to cancer via estrogen regulation, given estrogen's strong links to HR+ breast cancer, remains compelling. Improved measurement of the gut microbiome could clarify the estrobolome's role, but external factors such as diet, obesity, and physical activity might account for the lack of straightforward taxonomic relationships in existing studies.

Future studies would benefit from a broader view, as estrobolome mechanisms alone may not be sufficient to alter carcinogenic pathways. The microbiome functions as a complex network where immune and inflammatory mechanisms may also influence breast cancer risk. For instance, the gut microbiota can support immune regulation by producing short‐chain fatty acids that promote regulatory T cell differentiation and by stimulating gut‐associated lymphoid tissue to induce immunoglobulin A (IgA) secretion.^17^, ^84^, ^85^ Disruptions in these regulatory T cell and IgA pathways can increase inflammation, a risk factor for breast cancer.^86^ These pathways may independently affect breast cancer development or interact with estrobolome‐related mechanisms.

External factors may also shift the microbiome and influence estrobolome pathways, such as pro‐inflammatory diets,^87^ metabolic disorders (associated with increased inflammation),^88^ and aerobic exercise (associated with decreased chronic inflammation).^89^ To understand how theses external factors influence the estrobolome, studies need to capture a broader range of exposures, including dietary intake, physical activity, and prevalent diseases beyond breast cancer. Overall, our understanding of the estrobolome's role in breast cancer remains unclear, and it is uncertain whether estrogen‐specific mechanisms or broader ecological changes may play a more significant role in disease etiology.

## AUTHOR CONTRIBUTIONS

**Ashley H. Larnder:** Writing – original draft; writing – review and editing; conceptualization; investigation. **Amee R. Manges:** Supervision; writing – review and editing. **Rachel A. Murphy:** Writing – review and editing; supervision.

## CONFLICT OF INTEREST STATEMENT

The authors declare no conflicts of interest.
